# Improved Oxidation Stability of Camellia Oil-in-Water Emulsions Stabilized by the Mixed Monolayer of Soy Protein Isolate/Bamboo Shoot Protein Complexes

**DOI:** 10.3389/fnut.2021.782212

**Published:** 2021-12-01

**Authors:** Yuhang Xi, Aiping Zhang, Zhongjiang Wang, Shahzad Farooq, Cen Zhang, Liangru Wu, Hui Zhang

**Affiliations:** ^1^Zhejiang Key Laboratory for Agro-Food Processing, College of Biosystems Engineering and Food Science, Zhejiang University, Hangzhou, China; ^2^College of Food Science, Northeast Agricultural University, Harbin, China; ^3^Institute of Food Science, Zhejiang Academy of Agricultural Sciences, Hangzhou, China; ^4^China National Bamboo Research Center, Hangzhou, China; ^5^Ningbo Research Institute, Zhejiang University, Ningbo, China

**Keywords:** bamboo shoot protein, soy protein isolate, emulsion, complex, oxidation stability

## Abstract

The complex of soy protein isolate (SPI)/bamboo shoot protein concentrate (BPC) was developed to stabilize camellia oil-in-water (O/W) emulsions. The surface hydrophobicity of the BPC/SPI complex driven by hydrogen bonds and electrostatic attractions was improved. With the increasing ratio of BPC in the complex, a tighter network layer structure of the complex was formed due to the rearrangement of proteins, and the emulsions showed a progressive enhancement in the gel-like structures. At the SPI/BPC ratio of 2:1, the emulsions had smaller droplet size and lower creaming index of 230 nm and 30%, and the emulsifying activity and stability indices of the emulsions were 803.72 min and 11.85 g/m^2^, respectively, indicating a better emulsifying activity and stability of emulsions. Meanwhile, the emulsions stabilized by the complex at the ratio of 2:1 showed better storage and antioxidant stability. These findings are expected to develop the application of bamboo shoots in emulsion-based food products such as mayonnaise, salad dressings, and sauces.

## Introduction

Bamboo is a giant perennial arborescent grass that belongs to the family *Poaceae* containing over 1,250 species under 75 genera in the world (1). The bamboo shoot is the expanded bud or meristematic tissue of bamboo plants that grow into a tall bamboo plant within 3–4 months. Wang et al. (2) sorted out the protein of dozens of bamboo shoots and found that the highest protein level of the fresh bamboo shoots from D. *giganteus* and Y. *alpine* reached up to 3.86 g/100 g wb and 33.4 g/100 g db, respectively. Sayanika et al. (3) qualitatively analyzed the protein composition of bamboo shoots and found that the bamboo shoot had great potential as a healthy protein source for human consumption. Wang and Ng (4) extracted an antifungal protein (namely designated dendrocin) with a molecular weight of 20 kDa from the shoots of D. *latiflora Munro*, which showed a good inhibitory effect on mycelial growth. In addition, Liu et al. (5) evaluated antihypertensive and antihyperlipidemic effects of bamboo shoot angiotensin converting enzyme inhibitory peptide on high-fat-diet-induced rats, and found that the peptide showed improved antioxidant activity, which could serve as a latent inhibitor of angiotensin converting enzyme to prevent cardiovascular diseases.

Soy protein isolate (SPI) is a commercial soy protein product made from defatted soy flour by removing most of the non-protein components (e.g., fats and carbohydrates), and the protein content on dry basis in SPI is required to be over 90%. Owing to its amphiphilic characteristics and high surface hydrophobicity, SPI could serve as an excellent emulsifier to improve the emulsion stability (6). Liu and Tang (7) reported that the nanoparticle aggregates of soy proteins were obtained by heat treatments at 95°C for 15 min, which could form an effective pickering-like stabilizer for decreasing droplet size and increasing emulsion stability. Additionally, the addition of SPI could affect the rheology of emulsions, and then led to structural and functional changes, which may provide useful information for designing the emulsion-based food products such as mayonnaise, salad dressings, and sauces. Benetti et al. (8) produced the SPI microgel particles by ultrasound treatments and high pressure to modify the microstructure and rheological properties of the soybean oil-in-water (O/W) emulsions. Moreover, recent study showed that β-conglycinin and glycinin from SPI possessed good antimicrobial and antioxidant properties, which could improve the antioxidant activity of emulsions (9).

Protein-protein complexes can be formed by electrostatic attractive forces to improve emulsion stability. Compared with the emulsion stabilized by single protein, coadsorption of proteins at the oil-water interface could increase the viscosity and enhance the emulsion/foam stabilities. Wei et al. (10) prepared the emulsions stabilized by ovotransferrin-lysozyme complex through electrostatic interactions, and found that the emulsions had a decreased droplet size and displayed excellent stability during long-term storage. Zhang et al. (11) reported that the SPI/WPI complex could form an adsorption layer around the oil droplets, resulting in the improvement of emulsion stability.

The aim of this study was to probe the effects of the associative interactions between bamboo shoot protein concentrate (BPC) and SPI in the interfacial monolayer on physiochemical and rheological characteristics of the camelia O/W emulsions, which was expected to develop BPC as an additive to the emulsion-based food products. To that purpose, contact angle measurements, and Fourier transform infrared (FTIR) were used to characterize the physical properties of the SPI/BPC complex particles, respectively. Moreover, the droplet size and interfacial tension (IFT) of the emulsions were measured, and emulsifying activity index (EAI), emulsion stability index (ESI), and creaming index (CI) were used to evaluate the emulsion stability. In addition, rheological measurements were performed to study the viscoelastic properties and apparent viscosity. The peroxide value (POV) and thiobarbituric acid-reactive substances (TBARS) were measured to explore the antioxidant of the prepared emulsions. In addition, the microstructure of emulsion droplets was investigated by transmission electron microscope (TEM).

## Materials and Methods

### Materials

Soy protein isolated was purchased from Beijing OKA Biotechnology Co., Ltd. (Beijing, China). Camellia oil was bought from a local supermarket. Bamboo was provided by China National Bamboo Research Center. All the other chemicals were analytical grade.

### Extraction of BPC

Bamboo shoot protein concentrate was extracted from bamboo shoot following a previous method with some modifications (12). Briefly, the bamboo shoots were mixed with MilliQ water at 1:10 (w/v) and the pH was adjusted to 8.0 using 0.1 M NaOH solution. Subsequently, the mixture was constantly stirred in a water bath at 45°C for 2 h and then filtered with 80-mesh sieves. After centrifugation at 8,000 rpm for 25 min at 4°C, the supernatants were collected, and the precipitates were redissolved for centrifuge again. Then, BPC was obtained by spray drying and stored in a refrigerator at−20°C for further analysis.

### Preparation of SPI/BPC Complex and Emulsions

The SPI was dissolved in MilliQ water at a concentration of 4 wt%. BPC was added to the SPI solution with mass ratios of 0:1, 1:4, 2:4, 3:4, and 4:4 (wt/wt), respectively. To prepare SPI/BPC complexes, a certain amount of camelia oil (10% v/v) was added into the solution of SPI/BPC with the aqueous phase by continuously stirring, followed by the pH adjustment to 8.0 using 0.1 M NaOH solution. The mixture was further homogenized using a high-speed homogenizer (IKA T25 digital ULTRATURRAX®, Germany) at 15,000 rpm for 3 min. Afterward, the emulsions were prepared by high-pressure homogenization (Nano DeBEE30, BEE International, USA) at 25,000 psi for 5 min at 25°C.

### Three-Phase Contact Angle Measurements

The three-phase contact angle was measured on an OCA 20 contact angle meter (Data Physics Co. Ltd., Germany). Prior to measurement, the SPI/BPC complexes were pressed to the films with a thickness of about 2 mm. A syringe was used to transfer 3.5 μl of the solutions onto the surface, where the solutions were kept for 15 min. The contact angle was calculated by the SCA software.

### FTIR Spectroscopy

The infrared spectra of the particles were recorded on an FTIR instrument (Nicolet iS50, Thermo Fisher Scientific, China). The spectra were scanned with the absorbance mode of 4 cm^−1^ in the wavenumber range of 4,000–400 cm^−1^. All tests were conducted with air as the background.

The secondary structure of SPI and BPC was quantitatively determined by the analysis of the amide I region (1,700–1,600 cm^−1^) in the FTIR spectra. The contents of α-helix, β-sheet, β-turn, and random coil components were calculated using Peakfit 4.12 software (13).

### Droplet Size of Emulsions

The droplet size or diameter of the emulsions stabilized by SPI/BPC complex was measured by dynamic light scattering according to the method of Chantrapornchai et al. (14) with slight modification. Samples were diluted 1,000 times by the MilliQ water, and then the droplet size of the emulsions was measured on a Zetasizer Nano-ZS90 (Malvern Instruments, Worcestershire, UK). The refractive index of water and camellia oil were 1.33 and 1.462, respectively. All measurements were carried out at 25°C.

### Zeta Potential Measurement

The zeta potential of SPI/BPC emulsions was measured by the Zetasizer Nano-ZS90 (Malvern Instruments, Worcestershire, UK) at 25°C. Each sample was measured three times.

### Transmission Electron Microscope

The morphology characterization of emulsion droplets was performed using the TEM (Tecnai G2 Spirit, Thermo FEI, Czech Republic) at an accelerating voltage of 120 kV with the negative staining method (15). The samples (0.4 mg/ml) were added to a copper grid stained with phosphotungstic acid (1% w/v). The excess stain was removed, and the copper grids were dried at room temperature.

### IFT Measurements

The IFT of the emulsions was measured on a tensiometer (OCA 20, Data Physics Co. Ltd., Germany) with the pendant drop method. The droplet of distilled emulsion was suspended on the tip of a syringe (22 gauge, I-quip), and then the pictures of the droplet were captured by a video camera. The IFT values were calculated by Young-Laplace equation (16).


(2-1) $$\begin{array}{l} \gamma \left(\frac{1}{R_{1}}\;+\;\frac{1}{R_{2}}\right)\;=\;\Delta P \end{array}$$

where *R*_1_ and *R*_2_ are the principal radii of curvature, γ is the liquid surface tension coefficient, Δ*P* is the Laplace pressure across the interface.

### Emulsion Stability

#### Emulsifying Properties

About 100 μl of each emulsion sample was diluted with 5 ml of 0.1% (w/v) sodium dodecyl sulfate (SDS) solution and shaken rapidly. The absorbance was then measured at 500 nm.

Emulsifying activity index (m^2^/g) and ESI (min) were calculated using Equations (2–2) and Equations (2–3), suggested by Pearce and Kinsella (17):


(2-2) $$EAI\;=\;2\;\times \;2.303\;\times \;\frac{A_{0}\;\times \;d}{10000\;\times \;\Phi \;\times \;C}$$

(2-3) $$ESI\;=\;\frac{A_{0}}{A_{0}\;-\;A_{10}}\;\times \;(T_{10}\;-\;T_{0})$$

where *C* is the protein concentration (g/mL), Φ is the volume of the oil fraction (v/v) of the created emulsion (Φ = 0.1), *d* is the dilution factor (*d* = 500), *A*_0_ and *A*_10_ represent the absorbance at the beginning and after 10 min, respectively.

#### CI Measurements

Creaming index was calculated by a reported method (18). The freshly prepared emulsions were transferred into the same bottles and stored for 7 days at 25°C. CI was measured at 24 h intervals and calculated as follows:


(2-4) $$\begin{array}{l} CI\;=\;\frac{H_{S}}{H_{t}} \end{array}$$

where *H*_t_ is the total height of the emulsion, and *H*_s_ is the height of the creaming layer at the top.

### Rheological Properties

A strain sweep test was performed to determine the linear viscoelastic region from 0.01 to 100% at 1 Hz. The storage modulus (*G*′) and loss modulus (*G*″) of sample were measured by frequency sweeping from 0.1 to 100 Hz at a constant stain of 1% (19).

Viscosity was investigated as a function of shear rate on a rotational rheometer (MCR 302, AntonPaar, Graz, Austria), and the measurements were performed using a plate/plate measuring setup (PP25). The shear rate was increased from 0.1 to 100 s^−1^.

The model of Herschel-Bulkley was described by Eqs (2–5):


(2-5) $$\begin{array}{l} \sigma \;=\;\sigma_{0}\;+\;\kappa \;\times \;\gamma^{n} \end{array}$$

where σ (Pa) and σ_0_ (Pa) are the shear stress and the yield stress, respectively. *K* (Pa S^n^) is the consistency index, γ (s^−1^) represents the shear rate, and *n* represents the flow behavior index.

### Oxidative Stability of Emulsions

The POV was used to measure the primary oxidation products, which was detected using the method of Zhu et al. (20) with slight modification.

As an indicator of the secondary oxidation product, TBARS were evaluated using a previously reported method of Pan et al. (21) with slight modification.

### Statistical Analysis

All experiments were performed in triplicate with parallel samples. All the data were carried out by one-way ANOVA to evaluate significant differences (*p* < 0.05). Origin 9.0 software and SPSS18.0 were used for data analysis.

## Results and Discussion

### Three-Phase Contact Angle

**Figure 1:**
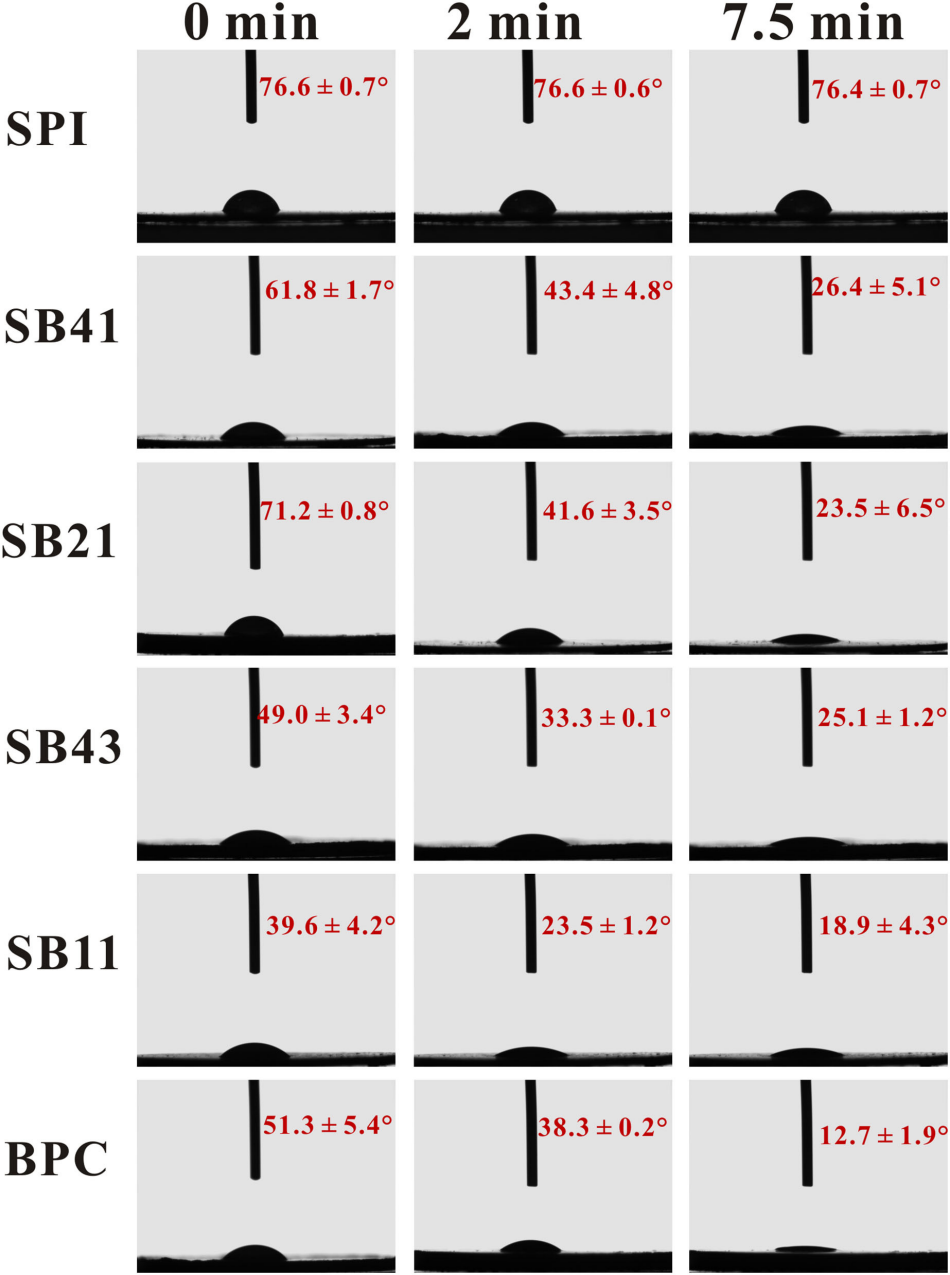
The three-phase contact angle of SPI, BPC, and SPI/BPC colloidal particles with different ratios (4:1, 2:1, 4:3, 1:1).

Pure SPI and BPC initially had the contact angle values of 76.6 and 51.3°, respectively, but the three-phase contact angle of BPC was reduced to 12.7° when standing for 7.5 min, while there was no obvious change of SPI in the three-phase contact angle. With the SPI/BPC ratio ranging from 4:1 to 2:1, the initial contact angle value of the complexes increased from 61.8 to 71.2° (Figure 1). This result might be attributed to the presence of the hydroxyl group in BPC, improving the surface hydrophobicity of the complexes by exposing the hidden hydrophobic area in the SPI (22). When the SPI/BPC ratio was 4:3 and 1:1, the contact angle of the complexes decreased to 49.0 and 39.6°, respectively, probably due to the presence of hydrophilic groups (such as OH group) carried by excessive BPCs (23). Previous results showed that the use of the particles with intermediate hydrophobicity as a stabilizer could improve the emulsion stability (24). Therefore, the SPI/BPC complex particles at 2:1 might have the best ability to stabilize the emulsions since the contact angle value was the closest to 90°.

### FTIR Analysis

**Figure 2:**
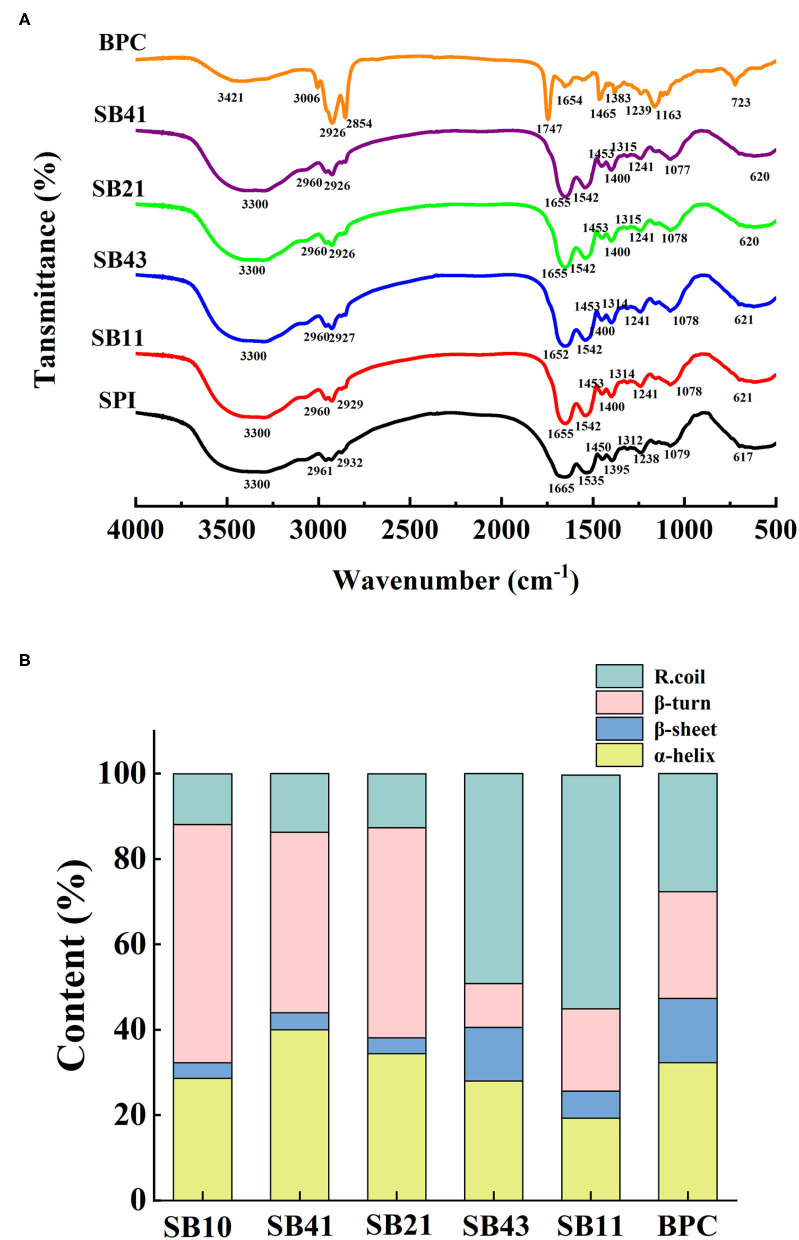
FTIR spectra **(A)**, secondary structure **(B)** of SPI, BPC, and different SPI/BPC ratios (4:1, 2:1, 4:3, 1:1).

Figure 2A shows the FTIR spectra of SPI and SPI/BPC particles at various mass ratios. There was a broad band over a wavenumber of 3,700–3,200 cm^−1^, associating with the free stretching vibration of hydroxyl groups in proteins (25). Compared with SPI, the band intensity of SPI/BPC complexes at about 3,300 cm^−1^ was enhanced, indicating hydrogen bonds formed between SPI and BPC. The peak positioned in the wavenumber range of 2,990–2,850 cm^−1^ represented the CH antisymmetric and symmetric stretching of methyl (CH_3_) and methylene (CH_2_) groups that appeared in aliphatic side chain of proteins, and then the peaks (2960 and 2926 cm^−1^) in the spectra were corresponding to the stretching (26). Moreover, the left shift of the C-N stretching and N-H bending vibration at 1,542 cm^−1^ was observed, which might be associated with the electrostatic attraction between the COO group in SPI and ${\text{NH}}_{3}^{+}$ group in BPC, thereby promoting the formation of SPI/BPC complexes (27).

As shown in Figure 2B, compared with BPC (45.7%), the β turns and random coil content of SPI/BPC particles from 4:1 to 1:1 increased from 63.5 to 71.2%, respectively, reflecting the formation of a more flexible structure that favored the adsorption and unfolding of protein molecules at the oil–water interface and then the enhanced emulsifying properties (13).

### Droplet Size

**Figure 3:**
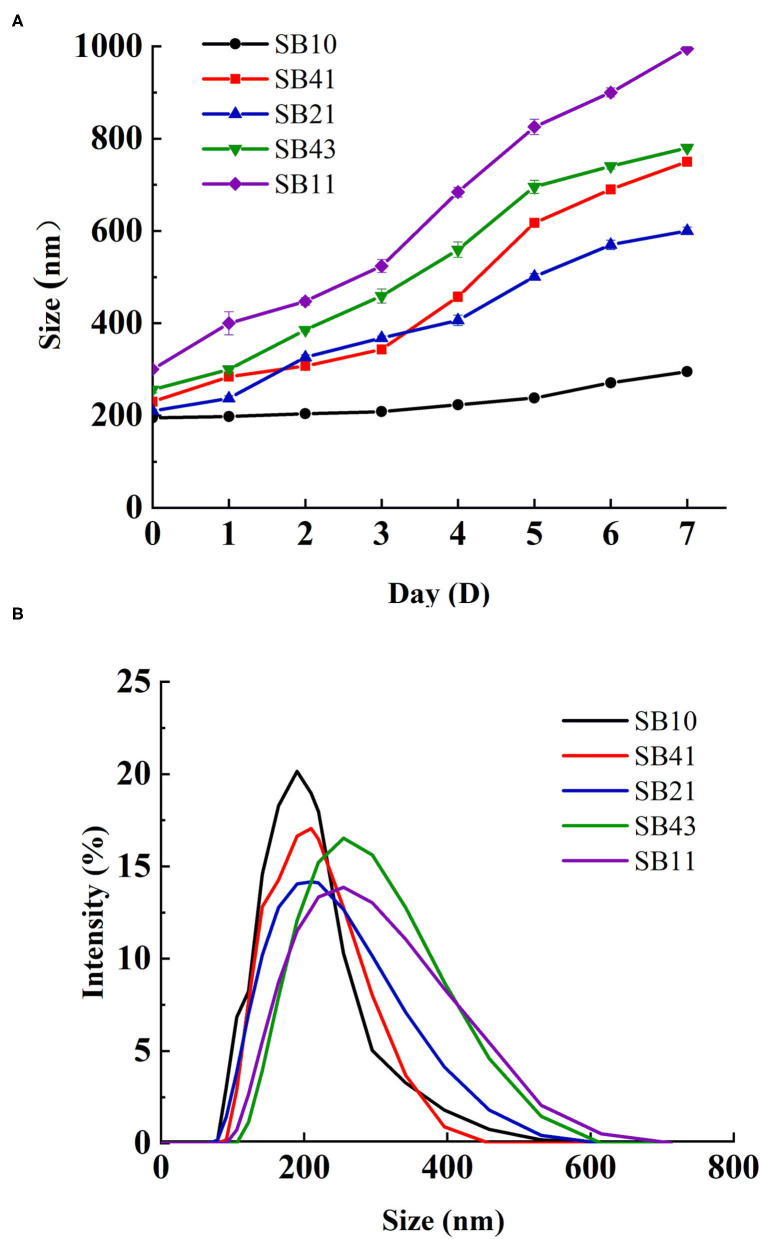
Changes of the emulsions in the average droplet size during storage for 7 days at 25°C **(A)**. Droplet size distribution of the emulsions **(B)**.

The mean size of the SPI-stabilized emulsions was about 200 nm and basically unchanged during 7 days of storage at 25°C (Figure 3A). However, the BPC-stabilized emulsions remained stable for only a few hours (data not shown). When the SPI/BPC complex at the mass ratio of 2:1 was employed as a stabilizer, the mean size of emulsion droplets increased from 200 to 595 nm during 7 days storage. As the mass ratio of BPC in the complex further increased, an increasing trend of the emulsions in droplet size was observed. Especially at the ratio of 1:1, the emulsions were inclined to separate after 7 days. These results indicated that a proper BPC concentration favored the faster adsorption of the complex on the water-in-oil interface, resulting in decreased IFT and then smaller droplet size (28). Moreover, the use of SPI/BPC complex at different ratios as stabilizers did not significantly affect the droplet size distribution of emulsions (Figure 3B), which agreed well with the previous reports of Wang et al. (29).

### Zeta Potential Analysis

The zeta potential of emulsions stabilized by SPI and SPI/BPC are shown in Table 1, and the absolute value of the zeta potential was used for comparison. When the mass ratio of BPC in the complex increased from 4:1 to 1:1, the zeta potential decreased significantly from −26.00 to −20.57 mV compared with the pure SPI stabilized emulsion, which was −38.13 mV, and this may be attributed to the hydrophobic interactions between SPI and BPC (30). Meanwhile, when the zeta potential decreased, the increase in the size of emulsions may be caused by the decreasing electrostatic repulsion and interfacial strength formed by SPI and BPC as the ratio of BPC increased (31).

**Table 1:** Zeta potential and interfacial tension of SPI, BPC, and different SPI/BPC ratios at various mass ratios.

**SPI/BPC ratio**	**Zeta potential (mV)**	**IFT (mN/m)**	**Vol (μL)**
1:0	−38.13 ± 0.95^a^	50.72 ± 0.10^a^	19.73 ± 0.23^a^
4:1	−26.00 ± 0.33^b^	50.25 ± 0.05^b^	19.09 ± 0.11^b^
2:1	−22.87 ± 0.29^c^	49.91 ± 0.11^b^	18.22 ± 0.23^c^
4:3	−19.33 ± 0.35^d^	49.16 ± 0.06^c^	16.89 ± 0.08^d^
1:1	−20.57 ± 0.19^d^	47.46 ± 0.14^d^	16.56 ± 0.13^e^

*Results are expressed as mean values ± SD of three replicates. Different letters in each column indicate significant difference (p < 0.05)*.

### TEM Analysis

**Figure 4:**
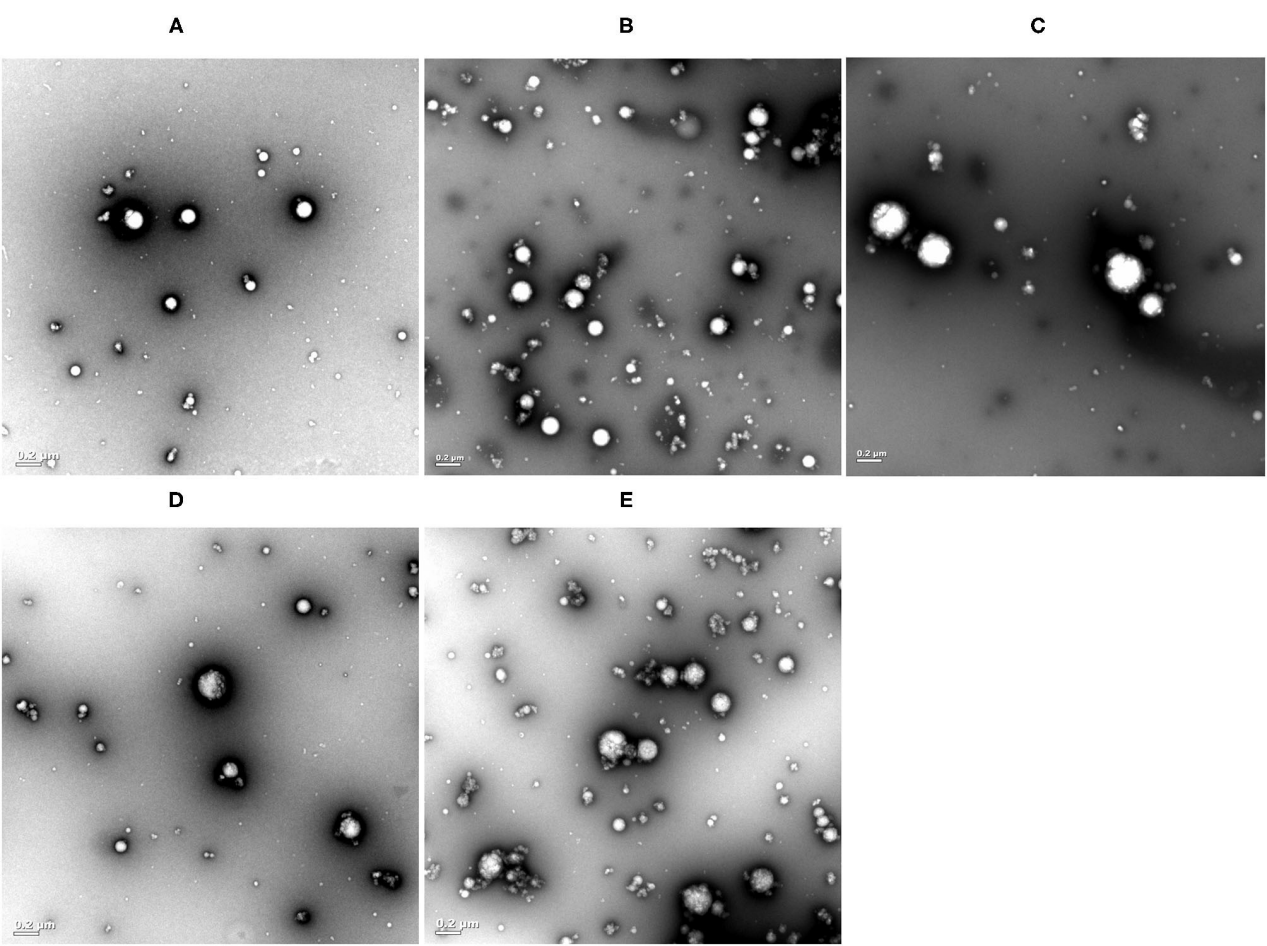
TEM images of the emulsions with different SPI/BPC ratios, SPI/BPC = 1:0 **(A)**, SPI/BPC = 4:1 **(B)**, SPI/BPC = 2:1 **(C)**, SPI/BPC = 4:3 **(D)**, SPI/BPC = 1:1 **(E)**.

The droplets of the pure SPI-stabilized emulsions possessed spherical shape and uniform size, with a diameter of approximately 200 nm (Figure 4), in agreement with the result of droplet size measurements. In the previous study, protein attached to the O/W interface could lead to a protein network structure (32). With the addition of BPC, the oil droplets were surrounded by SPI/BPC complexes, but the excess BPC may not absorb the oil-water interface, thus affecting the characteristic of the emulsions.

### IFT Analysis

The interfacial behavior of a stabilizer is considered an important factor influencing emulsion formation and stability. The reduced IFT and the mechanical energy barrier formed at the interface can prevent the emulsion system against coalescence (33). As shown in Table 1, SPI had the highest IFT value of 50.72 mN/m. With the increasing ratio of BPC in the complex, the IFT value of the complex decreased to 50.25 (4:1), 49.91 (2:1), 49.16 (4:3), and 47.46 (1:1) mN/m, respectively. According to Karaca et al. (34), the more soluble proteins favored to reduce IFT because the proteins could shift to the interface. This phenomenon has also been proven by Zhang et al. (35), who found that the IFT of all the four samples decreased as the SPI content increased, which helped more SPI molecules get adsorbed on the air-water interface.

### Emulsion Stability

#### ESI and EAI

**Figure 5:**
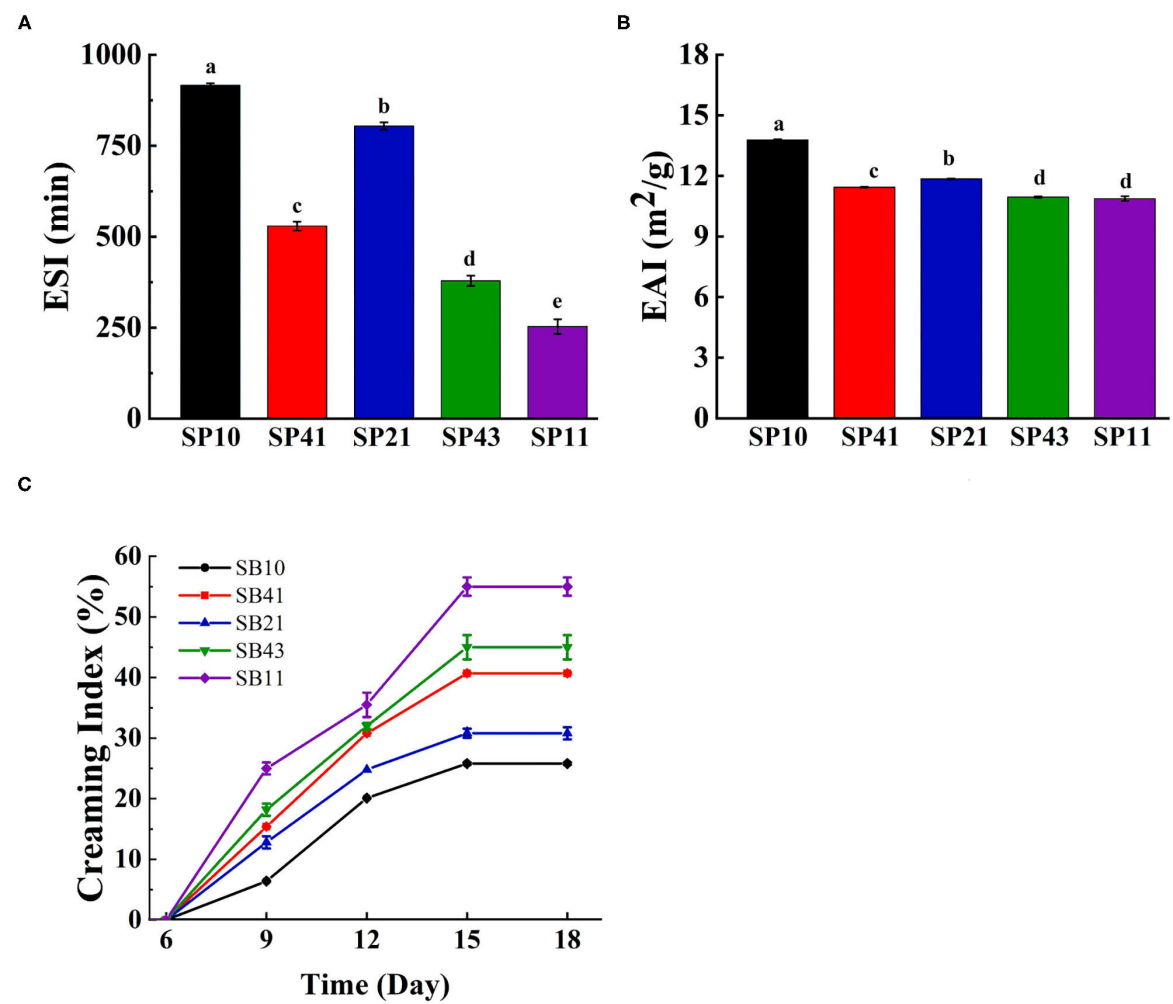
Emulsifying stability index (ESI) **(A)**, emulsion activity index (EAI) **(B)**, creaming index (CI) **(C)** of SPI, BPC and different SPI/BPC ratios (4:1, 2:1, 4:3, 1:1).

As shown in Figures 5A,B, the ESI and EAI of the emulsions at different ratios of BPC were significantly affected (*p* < 0.05), respectively. The emulsion stabilized by SPI exhibited the highest ESI (915.88 min) and EAI (13.79 g/m^2^). When the mass ratio of BPC in the complex increased from 4:1 to 2:1, the ESI and EAI of the emulsions increased from 529.15 to 803.72 min and from 11.44 to 11.85 g/m^2^, respectively, indicating that the addition of BPC could improve the emulsifying activity, and the oil droplets showed better dispersibility at a certain content of BPC (36). However, there was no significant difference in the EAI value between 10.95 and 10.87 g/m^2^ at the ratio of 4:3 and 1:1, indicating that excess BPC was freed from the interface to flocculate *via* hydrophobic interactions (37).

#### CI Analysis

In general, the CI and the size variation of the emulsion droplets during storage are used to characterize the emulsion stability (38). Figure 5C shows the curves of CI vs. storage time. No phase separation was observed in the freshly prepared emulsions (CI% = 0), which was maintained after 7 days of storage, except for the emulsion at the ratio of 1:1. The CI of all the emulsions increased with the increasing time of storage, but the emulsions remained stable until 18 days. As discussed under droplet size, when the SPI/BPC complex at the mass ratio of 2:1 was employed as a stabilizer, the mean emulsion droplet size decreased, resulting in lower creaming indices from 40.7 to 30%, which could be attributed to an insufficient content of stabilizers at the ratio of 4:1 to cover the oil droplets (39). The CI of emulsions increased from 30 to 55% when the ratio of SPI/BPC ranged from 2:1 to 1:1, which meant the creaming occurred more easily in the emulsions stabilized by the excess BPC and showed poor storage stability (40). Qamar et al. (41) also found a similar result that the CI increased from 5.77 to 8.39% as the concentration of pea protein increased from 0.5 to 1%, and the emulsion stability against flocculation and coalescence during storage might contribute to the creaming behavior of the pea protein emulsions.

### Rheology of Emulsions

**Figure 6:**
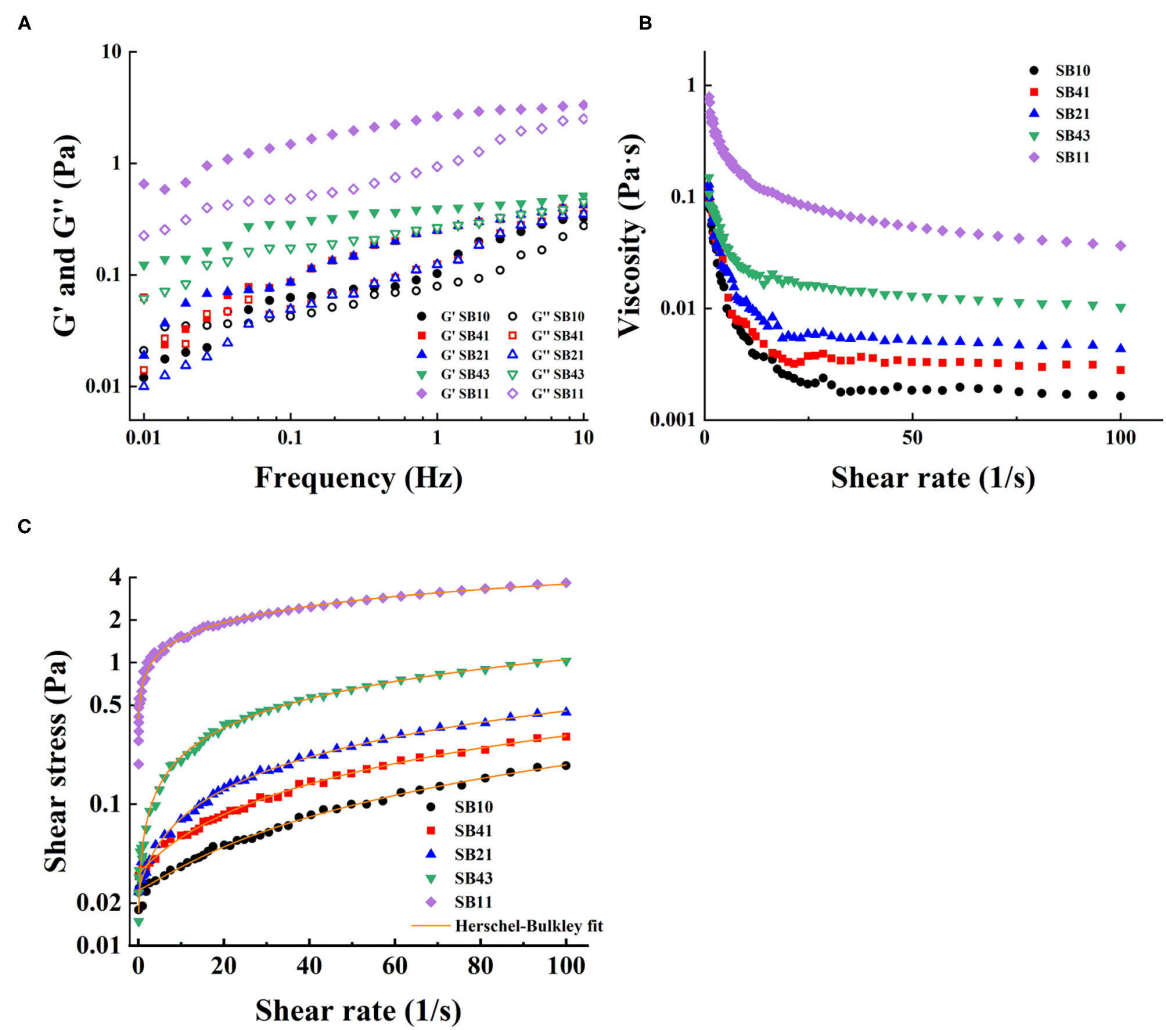
Dynamic frequency sweep of the emulsions **(A)**. Shear-rate dependence of the viscosity of the emulsions at various SPI/BPC ratios **(B)**. Plots of shear stress vs. shear rate for the emulsions. The solid lines are the lines of best fit to the Herschel-Bulkeley model **(C)**.

For all the emulsions, the *G*′ values were higher than *G*″ values at any given frequency (Figure 6A), suggesting the dominant elastic properties of the emulsions (42). Both *G*′ and *G*″ values of the emulsions at different SPI/BPC ratios increased with the increasing frequency ranging from 0.01 to 10 Hz, indicating that the modulus was frequency-dependent (43). In addition, with the ratio of BPC in the complex increased from 4:1 to 1:1, the *G*′ value was gradually increased to 3.66 Pa, suggesting a progressive enhancement in the gel-like structures (44).

The apparent viscosity (η) of the emulsions stabilized by SPI/BPC complex at various mass ratios gradually decreased with the shear rates increasing from 0.1 to 100 s^−1^ (Figure 6B), indicating a typical non-Newtonian pseudoplastic behavior. As the ratio of BPC increased from 1:0 to 1:1, the η value of the complex stabilized emulsions varied from 0.005 to 0.154 Pa·s at a shear rate of 10 s^−1^, which could lead to enhanced interaction between the droplets (44).

The Herschel–Bulkley model was used to evaluate the flow properties of the emulsions. As shown in Figure 6C, the shear stress vs. shear rate data at all the mass ratios showed a good correlation with Herschel–Bulkley models (*R*^2^ > 0.99). The *K* value increasing with the increased ratio of BPC in the complex reflecting the change in the viscosity of emulsion and the shear-thinning behavior of the emulsions as a non-Newtonian fluid. (45). Furthermore, the *n* values of all emulsion were <1 (Table 2), which confirmed the pseudoplastic fluid characteristics. The increased ratio of BPC in the emulsions decreased the *n* value, suggesting that the deviation from Newtonian behavior gradually increased (46).

**Table 2:** Rheological parameters of the emulsions stabilized by SPI/BPC complex using Herschel–Bulkley model.

**Mass ratios [SPI]: [BPC]**	**σ_0_ (Pa)**	***K* (Pa S^**n**^)**	***n***	***R* ^**2**^**
1:0	0.0247	0.008	1.167	0.994
4:1	0.0320	0.002	1.022	0.997
2:1	0.0231	0.007	0.888	0.998
4:3	0.0090	0.041	0.702	0.999
1:1	0.2232	0.478	0.424	0.996

### Oxidation Stability of Emulsions

**Figure 7:**
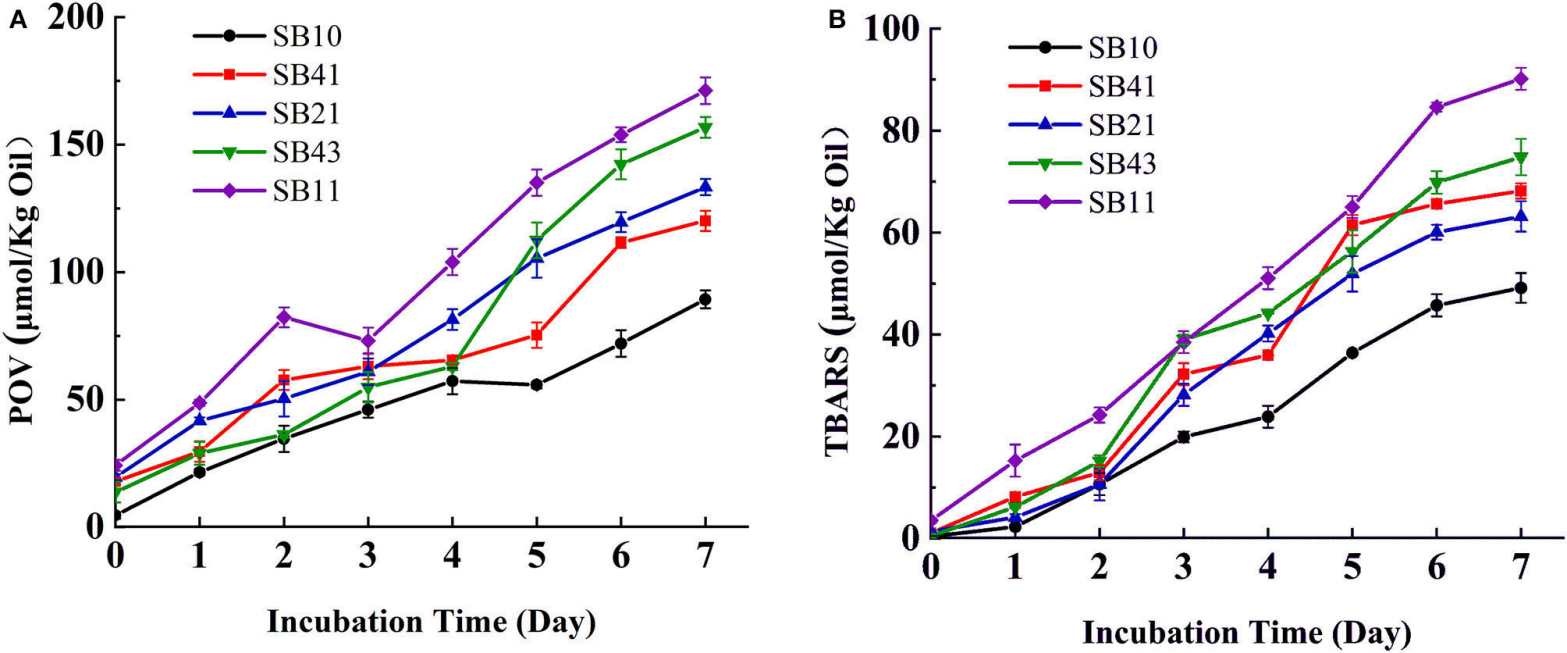
Changes in POV **(A)** and TBARS **(B)** of the emulsions with SPI, BPC, and different SPI/BPC ratios (4:1, 2:1, 4:3, 1:1), respectively.

Figure 7 shows the changes in POV and TBARS of the emulsions as a function of storage time at 25°C. POV indicates the levels of hydroperoxides formed from fatty acid radicals during oxidation processes, while TBARS shows the content of malondialdehyde that is a secondary lipid oxidation product from polyunsaturated fatty acids (47). After 7 days of storage, the POV value of the emulsions stabilized by the SPI/BPC complex at the mass ratio of 1:1 increased remarkably from almost zero to 171 μmol/kg oil, revealing the occurrence of lipid oxidation in the emulsions during storage. In addition, the POVs of emulsions increased from 89.3 to 171 μmol/kg oil with the ratio of SPI/BPC increasing from 4:1 to 1:1. The changes in TBARS contents had a similar trend to those of POVs, indicating that the primary oxidation products might be transformed into secondary oxidation products (48). Generally, emulsion system trended to be oxidized due to the existence of an oil–water interface, and some unabsorbed molecules could affect the lipid oxidation process like the excess BPC content in the ratio of 1:1 (49).

## Conclusions

In this study, the camellia O/W emulsions were prepared with the SPI/BPC complex as a new stabilizer. The hydrogen bonding and electrostatic attractions dominated the binding of BPC to SPI, which could improve the surface hydrophobicity and induce a network layer structure of the SPI/BPC complex at the mass ratio of 2:1. When the complex was used to stabilize the emulsions, the increasing ratio of BPC increased the droplet size but decreased the IFT. The emulsions had a shear-thinning and dominant elastic behavior, and the emulsions stabilized by the complex at the ratio of 2:1 showed better storage and antioxidant stability. This work showed the great potential of BPC as a new additive to the emulsion-based food products.

## Data Availability Statement

The raw data supporting the conclusions of this article will be made available by the authors, without undue reservation.

## Author Contributions

YX: conceptualization, methodology, investigation, data curation, and writing-original draft preparation. AZ: formal analysis and software. ZW: resources and investigation. SF: formal analysis and data curation. CZ: methodology, data curation, and supervision. LW: validation and visualization. HZ: project administration, visualization, conceptualization, review and editing, and funding acquisition. All authors contributed to the article and approved the submitted version.

## Funding

This study was financially supported by the Zhejiang Provincial Natural Science Foundation of China for Distinguished Young Scholars (Grant No. LR20C200001), and the Zhejiang Provincial Natural Science Foundation of China (Grant No. 2020C02036 and 2021C02032).

## Conflict of Interest

The authors declare that the research was conducted in the absence of any commercial or financial relationships that could be construed as a potential conflict of interest.

## Publisher's Note

All claims expressed in this article are solely those of the authors and do not necessarily represent those of their affiliated organizations, or those of the publisher, the editors and the reviewers. Any product that may be evaluated in this article, or claim that may be made by its manufacturer, is not guaranteed or endorsed by the publisher.
